# Access to Treatment for Hepatitis B Virus Infection — Worldwide, 2016

**DOI:** 10.15585/mmwr.mm6728a2

**Published:** 2018-07-20

**Authors:** Yvan Hutin, Muazzam Nasrullah, Philippa Easterbrook, Boniface Dongmo Nguimfack, Esteban Burrone, Francisco Averhoff, Marc Bulterys

**Affiliations:** ^1^Department of HIV and Global Hepatitis Programme, World Health Organization, Geneva, Switzerland; ^2^Division of Viral Hepatitis, National Center for HIV/AIDS, Viral Hepatitis, STD and TB Prevention, CDC; ^3^Medicines Patent Pool, Geneva, Switzerland.

Worldwide, an estimated 257 million persons are living with chronic hepatitis B virus (HBV) infection (*1*). To achieve the World Health Organization (WHO) goals for elimination of HBV infection worldwide by 2030, defined by WHO as 90% reduction in incidence and 65% reduction in mortality, access to treatment will be crucial. WHO estimated the care cascade* for HBV infection, globally and by WHO Region. The patent and licensing status of entecavir and tenofovir, two WHO-recommended medicines for HBV treatment, were examined using the Medicines Patent Pool MedsPaL^†^ database. The international price of tenofovir was estimated using WHO’s global price reporting mechanism (GPRM), and for entecavir from a published study (*2*). In 2016, among the estimated 257 million persons infected with HBV worldwide, approximately 27 million (10.5%) were aware of their infection, an estimated 4.5 million (16.7%) of whom were on treatment. In 2017, all low- and middle-income countries (LMICs) could legally procure generic entecavir, and all but two LMICs could legally procure generic tenofovir. The median price of WHO-prequalified generic tenofovir on the international market fell from $208 per year in 2004 to $32 per year in 2016. In 2015, the lowest reported price of entecavir was $427 per year of treatment (*2*). Increased availability of generic antivirals effective in treating chronic HBV infection has likely improved access to treatment. Taking advantage of reductions in price of antivirals active against HBV infection could further increase access to treatment. Regular analysis of the hepatitis B treatment care cascade can assist in monitoring progress toward HBV elimination goals. 

Hepatitis B vaccination among infants has increased globally (*3*); in 2015, the prevalence of chronic HBV infection in children aged <5 years was estimated at 1.3% (*1*). However, HBV infection remains prevalent among adults, with an estimated 257 million persons (3.5% of the population) living with chronic HBV infection worldwide in 2015 (*1*). Persons with chronic HBV infection are at increased risk for cirrhosis and hepatocellular carcinoma, and nearly 900,000 persons die annually from HBV-related outcomes, primarily from these sequelae of infection (*1*).

In 2015, WHO issued guidelines for the management of chronic HBV infection, particularly for LMICs (*4*). The available medications suppress viral replication and can decrease mortality (*4*), but the need for treatment is potentially lifelong. Access to treatment is limited in LMICs, because of lack of awareness among patients, cost and availability of quality diagnostics, cost of medicines, and lack of trained health care providers (*1*). In 2016, the World Health Assembly endorsed WHO viral hepatitis elimination goals, defined as a 90% reduction in incidence and a 65% reduction in mortality worldwide for both hepatitis B and hepatitis C by 2030 (*1*). To reach the mortality reduction goals for HBV elimination, a major scaling up of treatment will be needed. This report assesses global progress in access to hepatitis B treatment in 2016 (*1*).

WHO described the sequential stages of hepatitis B care that persons living with HBV infection go through from diagnosis to viral suppression (the care cascade) (*5*). The 2015 WHO estimates of the number of persons infected with HBV were used as the denominator (*1*). In each country, the estimated size of the population infected with HBV, the number of persons treated, the proportion of infected persons who had received a diagnosis, and the proportion treated were obtained from published estimates (*6*). Estimates of the size of the population that had received a diagnosis of HBV infection were derived from the following data sources (in order of priority): 1) national notifications; 2) published studies; 3) blood donation screening data; and 4) extrapolations from neighboring countries. The number of persons treated for HBV infection was estimated using the following data sources (in order of priority): 1) national reports; 2) sales audit data of generic tenofovir; 3) published studies; and 4) national experts’ estimates. To use medicine sales audit data, the annual number of treatment units sold were converted into the number of treated patients using the average number of units per patient. Assuming 90% viral suppression among patients with full adherence (*1*,*6*) and 80% treatment adherence, it was estimated that globally, 72% achieved effective viral suppression (*1*,*6*). The country cascade estimates were weighted by population size. Data from all regions were summed to generate a global estimate that was stratified by WHO region^§^ and four World Bank income groups.^¶^

The patent and licensing status of entecavir and tenofovir, the two WHO-recommended HBV medications, influence medicine prices (*7*). The relevant U.S. patents of medicines were identified from the Food and Drug Administration Orange Book (*8*). Information on the patent status was then collected from the relevant offices, and the global coverage of tenofovir with voluntary licenses was analyzed using data obtained from the Medicines Patent Pool’s database, MedsPaL.

The international price of tenofovir was estimated using WHO’s GPRM,** which reports market price of WHO-prequalified commodities used for human immunodeficiency virus (HIV) response based on transactions reported by major purchasers (e.g., the President's Emergency Plan for AIDS Relief [PEPFAR], the Global Fund, South Africa, United Nations Children’s Fund [UNICEF]). Because the GPRM does not monitor the price of entecavir, which is not a first-line HIV medicine, the price reported in a 2015 study was used to estimate the price of this medicine (*2*).

Among the estimated 257 million persons infected with HBV worldwide, an estimated 27 million (10.5%) had received a diagnosis and were aware of their infection in 2016, 4.5 million (16.7%) of whom were estimated to be receiving HBV treatment (Figure 1). Treatment coverage was low among countries in all income strata, but highest (22%) in upper middle-income countries (Figure 2). In 2017, all LMICs could legally procure generic entecavir, while all but two LMICs (China and Mexico) could legally procure generic tenofovir. However, the relevant patents in China and Mexico are set to expire in 2018. Tenofovir patents in the United States and Europe also expired in 2017 and 2018. The median price of WHO prequalified generic tenofovir available on the international market fell by over 85%, from $208 per year of treatment in 2004 to $32 in 2016 (Figure 3), in tandem with expanding access to tenofovir-based antiretroviral treatment regimens for persons living with HIV infection. In 2015, the lowest reported price of entecavir was $427 per year of treatment (*2*).

**FIGURE 1:**
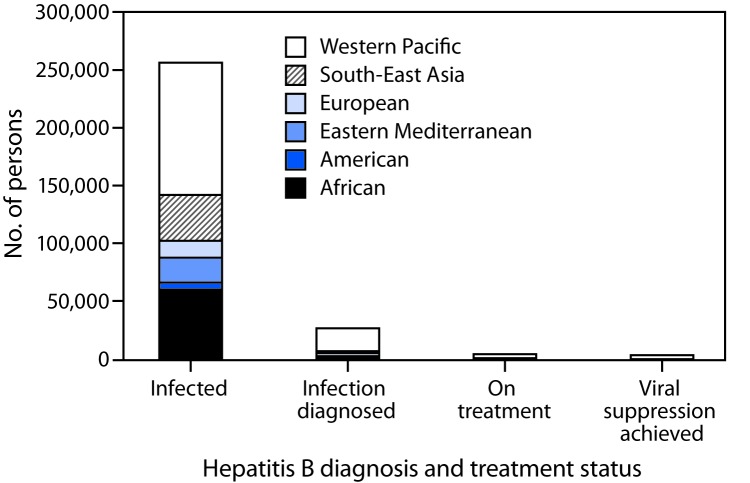
Care cascade* for hepatitis B treatment, by World Health Organization region, 2016 * The sequential steps or stages of hepatitis B care that persons living with hepatitis B virus infection go through, from diagnosis through viral suppression.

**FIGURE 2:**
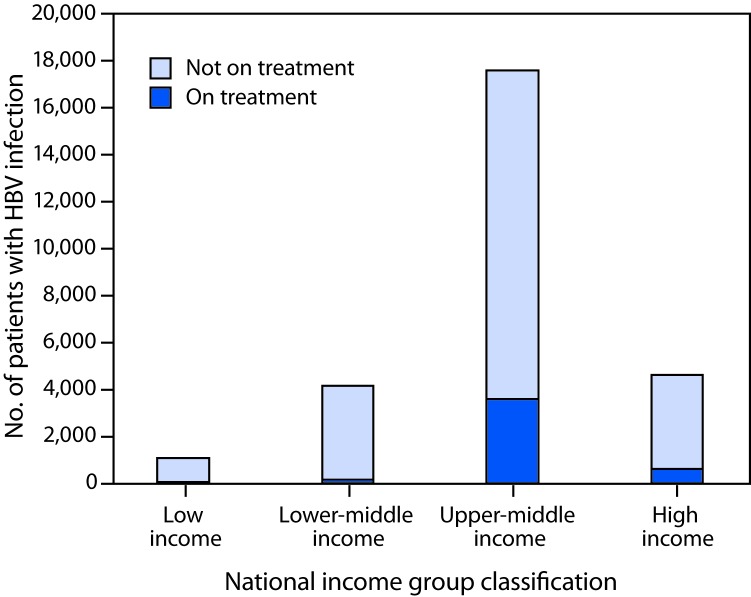
Hepatitis B virus (HBV) treatment coverage among the 27 million persons with diagnosed HBV infection, by national income group — worldwide, 2016

**FIGURE 3:**
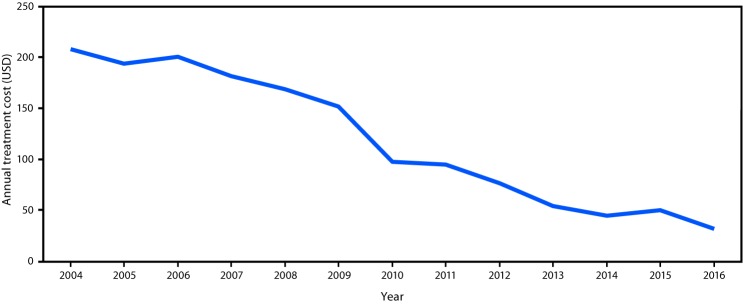
Reported annual cost of treatment for hepatitis B virus infection with tenofovir in countries that can access generic medicines — worldwide, 2004–2016 **Source:** World Health Organizations Global Price Reporting Mechanism.

## Discussion

In 2016, only one in 10 persons living with HBV infection worldwide had received a diagnosis, and among those, one in six was receiving treatment. Treatment coverage varied by WHO region and by national income group and was highest in the Western Pacific region and upper middle-income countries. Price reduction for antiviral medicines and inclusion of the treatment in the various health insurance packages in China (*1*) probably led to a major increase in the number of persons treated, which likely accounts for the higher treatment coverage in this region. Africa has among the highest prevalence of HBV infection and highest mortality from liver cancer in the world (*1*). However, in the African region, with the exception of a few demonstration projects (*9*), and for persons with HIV-HBV coinfection who are on antiretroviral regimens (current antiretroviral treatment regimens include medications active against HBV infection), no national programs are known to provide testing, care, and treatment services for all persons with HBV infection.

Although the price of medicines effective against HBV infection have decreased sharply in LMICs, the findings from this analysis indicate underutilization of low-price, generic medicines effective against HBV. Greater community awareness and better understanding of the national disease burden, access to and availability of affordable diagnostics, and trained providers are needed to promote increased access to care.

The findings in this report are subject to at least five limitations. First, WHO-endorsed global estimates of the proportion of patients with HBV infection eligible for treatment (not all HBV infected persons should be on treatment, based on disease stage) are not available (*4*); therefore, it was not possible to estimate how many more persons currently require treatment in addition to the 4.5 million already receiving treatment. Second, the estimates of the number of persons on treatment are based on data sources of variable quality, and the results needed to be generalized by region/country when data were not available. Third, methods used to obtain information on the price of entecavir and tenofovir came from different sources. However, the published study that estimated the price of entecavir reported prices for tenofovir that were comparable with the data obtained from the GPRM. Fourth, it was not always possible to determine whether tenofovir was prescribed as part of HIV-related antiretroviral regimen or for HBV infection alone. Finally, no systematic mechanism is available to monitor the prices of HBV medicines sold in the private sector.

Development and use of the global HBV care cascade can assist in monitoring progress toward the WHO 2030 HBV elimination goals. Refinements in the care cascade to account for the proportion of HBV-infected persons who are not eligible for treatment could improve estimate precision. Increased access to generic HBV medicines through price reductions might lead to a larger proportion of HBV-infected persons receiving treatment. Development of a national viral hepatitis control strategy is an important step for increasing access to treatment for persons with chronic HBV infection, particularly in countries with a large disease burden (*1*). Development of such strategies might open the way to public sector procurement of medicines and diagnostics that can lower prices. Among patients with HBV infection, those with cirrhosis should be prioritized for treatment, because they are at increased risk for developing life-threatening complications. WHO is in the process of establishing a reporting system to monitor the cascade of care globally to allow for regular reporting. The potential future availability of a functional cure for HBV infection will further improve the prospects of achieving the 2030 elimination targets (*10*).

**Summary**

**What is already known on the topic?**

An estimated 257 million persons were living with chronic hepatitis B virus (HBV) infection in 2015.

**What is added by this report?**

Among persons living with HBV worldwide, approximately 27 million (10.5%) were aware of their infection, including 4.5 million (16.7%) who were on treatment. In 2017, all but two low- and middle-income countries could legally procure generic entecavir or tenofovir, the medicines active against HBV infection. The median price of generic tenofovir fell by >85% from 2004 to 2016. However, global treatment coverage of HBV was low.

**What are the implications for public health practice?**

Access to treatment could be increased by taking advantage of reductions in price of antivirals active against HBV infection.

## Footnotes

* The sequential steps or stages of hepatitis B care that persons living with hepatitis B virus infection go through, from diagnosis through viral suppression.

†
http://www.medspal.org .

§ Africa, Americas, Eastern Mediterranean, Europe, South-East Asia, and Western Pacific.

¶ Low-income countries; lower, middle-income countries; upper, middle-income countries; and high-income countries.

** http://apps.who.int/hiv/amds/price/hdd/; cost data was only available for tenofovir.
